# Epidemiology of pre-pregnancy body mass index (BMI) among mothers in Abu Dhabi, the United Arab Emirates

**DOI:** 10.3389/fgwh.2022.893808

**Published:** 2022-09-13

**Authors:** Zainab Taha, Ahmed Ali Hassan, Dimitrios Papandreou

**Affiliations:** ^1^College of Natural and Health Sciences, Zayed University, Abu Dhabi, United Arab Emirates; ^2^Abu Dhabi Public Health Centre, Abu Dhabi, United Arab Emirates

**Keywords:** pre-pregnancy BMI, overweight, obesity, cesarean section, birth order, United Arab Emirates

## Abstract

Pre-pregnancy obesity is a risk factor for several health problems such as gestational diabetes, preeclampsia, labor induction, postpartum hemorrhage, and neonatal hypoglycemia. Being underweight is a risk factor for fetal growth restriction. Despite the negative impact of abnormal pre-pregnancy body mass index (BMI) (over and underweight) on pregnancy outcomes, a limited amount of studies has been conducted on the prevalence of pre-pregnancy over and underweight and associated factors in the United Arab Emirates (UAE). Thus, the aim of this study was to investigate the prevalence and factors associated with underweight, overweight, and obesity among mothers with children under the age of 2 years in Abu Dhabi, the UAE. A cross-sectional multicenter study was conducted in 2017 and included data from seven government health care centers in Abu Dhabi. Maternal pre-pregnancy BMI was calculated as the weight in kilograms (kg) divided by the square of the height in meters (m), (kg/m^2^). A total of 1,622 mother–child pairs were included in this study. Out of the 1,622 mothers, 43 (2.7%) were underweight, 1,068 (65.8%) were normal weight, 412 (25.4%) were overweight, and 99 (6.1%) were obese. Mothers with advanced maternal age (AMA) (≥35 years) were more likely to be overweight, odds ratio [OR] = 1.93, (95% confidence interval [CI] 1.52–2.3), *p* ≤ 0.001, and obese OR = 2.15 (95% CI 1.32–3.39), *p* = 0.001. Mothers with lower family income were more likely to be obese OR = 2.72 (95% CI 1.44–5.93), *p* = 0.002. Mothers with high parity (≥2) were more likely to be overweight OR = 1.91 (95% CI 1.49–2.50), *p* ≤ 0.001; obese OR = 1.76 (95% CI 1.06–2.92), *p* = 0.024; and less likely to be underweight OR = 0.52 (95% CI 0.27–0.94), *p* = 0.037. Obese mothers were more likely to deliver via cesarean section (CS) OR = 1.95 (95% CI 1.27–2.96), *p* = 0.002. This study provides valuable baseline information on the epidemiology of pre-pregnancy BMI in the UAE. The sociodemographic factors identified in the study can be used to target at-risk women. The study findings can also be used to develop contextualized strategies to prevent and manage complications associated with abnormal pre-pregnancy BMI in Emirati women.

## Introduction

In recent years, the prevalence of overweight and obesity has been dramatically increasing worldwide, including women of reproductive age as well as pregnant mothers (1). In the United Arab Emirates (UAE), a systematic review assessing the prevalence of overweight and obesity prevalence reported a 2–3-fold increase between 1989 and 2017 (2). These results should be considered alarming because the obesity rates were higher among women, including women of reproductive age and pregnant women (42%) (3).

A previous study has documented that pre-pregnancy BMI has a more significant impact on pregnancy outcomes than weight gain during pregnancy (4, 5). Several studies addressed the effects of maternal pre-pregnancy obesity on pregnancy outcomes, including maternal and newborn health, such as gestational diabetes, gestational hypertension, macrosomia (6–8), preterm delivery (9–11), delayed initiation of breastfeeding, and a shortened duration of exclusive breastfeeding (12).

Before pregnancy, being underweight was also found to be associated with adverse health issues related to newborn health, such as the delivery of low birth weight (LBW) infants (13, 14). In addition, obesity, as well as overweight, may also affect the mode of delivery, which ultimately may have an impact on healthcare costs and maternity services (15). Concurrent with the rise in obesity prevalence, the rate of cesarean section (CS) has increased in the UAE from 10% in 1995 (16) to 24% in 2014 (17). A recent study reported a higher CS rate in Abu Dhabi at 30.2% (18). These rates are significantly higher than those suggested by the World Health Organization (WHO), of 10–15% of all live births (19). They are alarming when their impact on maternal and infant health is considered. Several studies reported an increased risk of CS in obese women (20–22).

In view of the abovementioned health problems, an important research area should also consider the sociodemographic characteristics that affect pre-pregnancy body mass index (BMI). Identifying the sociodemographic factors associated with pre-pregnancy BMI and maternal obesity is useful for implementing targeted preventive actions and improving their efficacy. However, the results appear inconsistent across the studies that have already investigated the relationship between sociodemographic factors and pre-pregnancy BMI. For example, some studies identified poor socioeconomic conditions, based on the level of the family income as low or living in deprived conditions to be significantly associated with maternal obesity (5, 23, 24). Other researchers of low-income countries found that maternal underweight in early pregnancy is most probably related to pre-pregnancy underweight as a leading risk factor for adverse birth outcomes, including LBW, preterm birth, and small for gestational age (25, 26).

However, there have been inconsistent findings for other sociodemographic factors such as maternal age and education level (5, 23, 27). An understanding of the sociodemographic factors associated with abnormal pre-pregnancy BMI can be useful in designing appropriate measures. There is not only a lack of studies from the UAE on the epidemiology of pre-pregnancy BMI but also on associated sociodemographic factors. Therefore, we undertook this study to understand the prevalence of abnormal pre-pregnancy BMI and the associated sociodemographic factors.

## Materials and methods

### Participants and data collection

This study was a multicenter cross-sectional study where mothers of children were recruited from the community and healthcare centers in Abu Dhabi from March to September 2017. The study sample included Emirati and non-Emirati families. Seven governmental clinics providing maternal and child health services were approved for data collection. Mothers with young children visiting the healthcare centers with their children were approached by trained female research assistants who provided oral and written information about the study in Arabic and English. The inclusion criteria for recruiting participants had at least one child under 2 years of age. The research assistants interviewed the mothers using a structured questionnaire for data collection. The original project from which these data were extracted was approved (ZU17_006_F) by the Research Ethics Committee at Zayed University, the UAE. Also, clearance was obtained from the Abu Dhabi Health Services Company.

### Study instrument

The primary study tool was a questionnaire validated by conducting a pilot study where necessary corrections were done before the distribution to collect the data. The questionnaire was first designed in English and then interpreted in Arabic, using a cross-translation strategy, where a local Arabic speaker translated the English document into Arabic. After that, another local Arabic speaker blinded to the original translation and converted the document back to English. The questionnaire included family demographics (e.g., parent education, age, nationality, and occupation, maternal and paternal education, occupation, maternal age, family financial status), child's information, health indicators (e.g., child gender, birth weight, delivery mode, parity), and infant feeding practices (e.g., breastfeeding initiation).

More information about the study methodology was described in the previous study (28).

### Variables

The primary variable of this study is pre-pregnancy BMI (underweight, normal weight, overweight, and obese). The secondary variables are sociodemographic characteristics such as maternal age in years, parity, parent education, occupation; pregnancy outcomes such as gestational age at delivery (term vs. preterm), birth weight at delivery (normal birth weight vs. LBW), and mode of delivery (vaginal vs. CS). These variables are selected based on previous studies (5, 23–27). For each variable, odds ratio (OR) and 95% CI was calculated, and any variable with a *p* < 0.05 was considered significant.

### Definitions

#### Education

Education is categorized into less than secondary and secondary and above, i.e., < secondary education is considered primary education, unlike ≥ secondary education level.

#### Body mass index (BMI)

BMI is defined as the weight in kilograms divided by the square of the height in meters (kg/m^2^). Both pre-pregnancy maternal weight and height were provided from the maternal health cards. The BMI was categorized into subgroups using the WHO classification (29) as follows: underweight (BMI <18.5 kg/m^2^), normal weight (BMI 18.5–24.9 kg/m^2^), overweight (BMI 25–29.9 kg/m^2^), and obese (BMI ≥ 30 kg/m^2^).

#### Parity

Number of times that a woman had given birth to a fetus with a gestational age of 24 weeks or more, regardless of whether the child was born alive or was stillborn.

#### Gestational age (GA) at delivery

A measure of the age of a pregnancy in weeks, which is taken from the beginning of the woman's last menstrual period. Term birth was defined as the birth of a baby at ≥37 weeks GA. Preterm birth was defined as the birth of a baby at <37 weeks GA.

#### Birth weight at delivery

Refers to the baby's weight in grams immediately after delivery; any baby delivered with a weight <2,500 grams was considered as LBW and ≥2,500 grams as normal weight.

#### Arab nationality

All Emirati mothers and other Arab nationalities.

#### Non-Arab nationality

Asian mothers and other non-Arab nationalities.

#### Family income

Based on the mother's answer to the following question, “Considering your monthly family income, how would you rate your and your family's overall financial well-being?” < good or ≥good.

### Data analysis

Data were analyzed using Statistical Package for the Social Science (IBM SPSS Statistics for Windows, Version 20.0. Armonk, NY: IBM Corp.). Descriptive statistics were applied to estimate the prevalence with a 95% confidence interval (CI) among different pre-pregnancy BMI groups. Means and proportions for the sociodemographic characteristics were compared between the four subgroups of the pre-pregnancy BMI (underweight, normal, overweight, and obese) by ANOVA and Chi-square tests, respectively. Continuous variables, such as maternal age, parity, gestational age, and birth weight at delivery, were also analyzed as continuous and categorical variables. As the dependent variable (pre-pregnancy BMI) comprised four subgroups, multi-nominal logistic regression was applied. In the ANOVA and Chi-square tests, significant variables (*p* < 0.05) were further analyzed by logistic regression.

## Results

**Figure 1:**
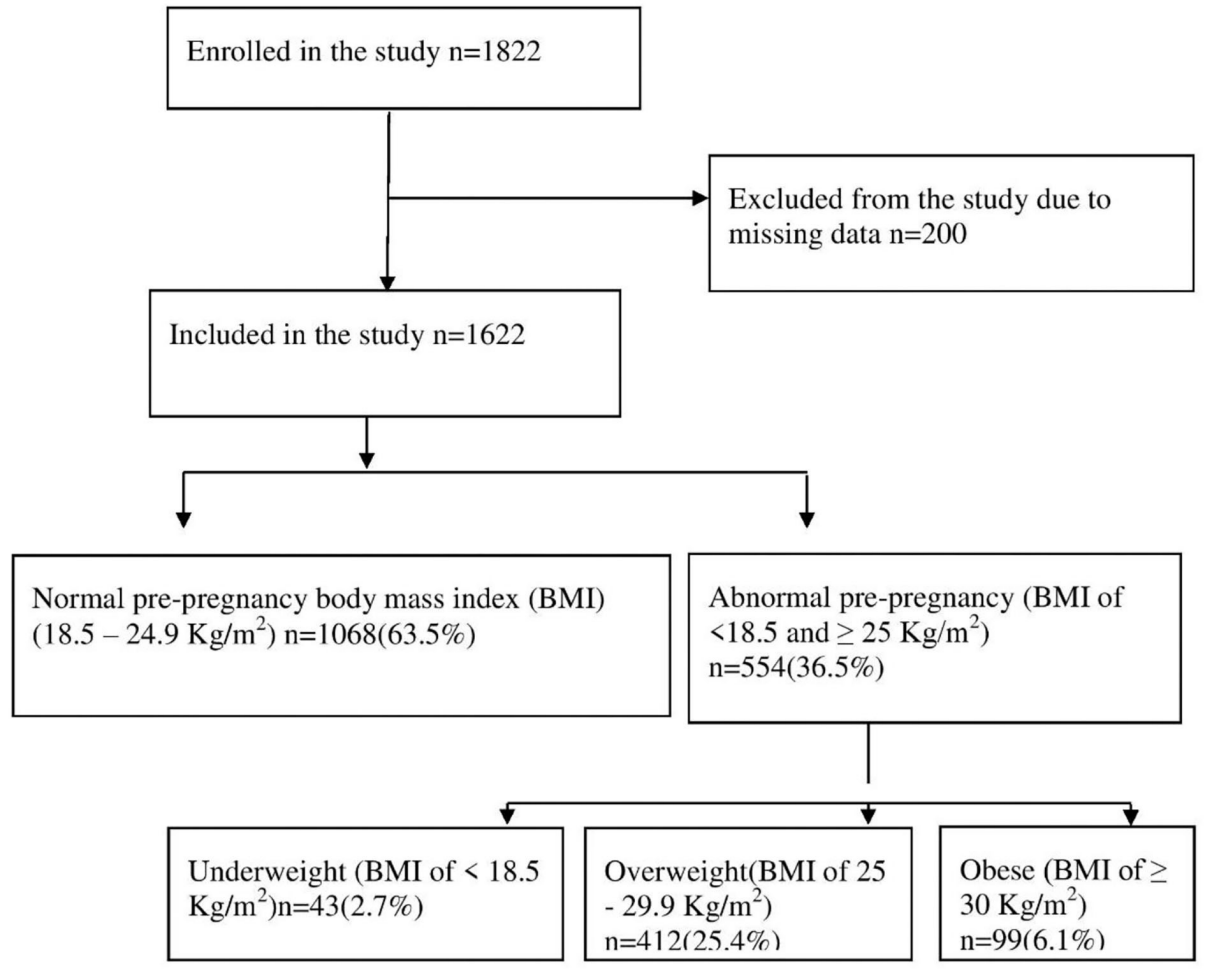
Flowchart of the study participants and the main findings.

A total of 1,622 mother–child pairs were included in this work from the original sample (*N* = 1,822). The remaining 200 participants were excluded due to missing data such as maternal education, gestational age, and mode of delivery (Figure 1).

The mean (SD) of maternal age and parity was 30.1 (5.2) years and 2.2 (1.2), respectively. Out of 1,622 mothers, 43 (2.7%) (95% CI 2.5–2.9) were underweight (BMI <18.5 kg/m^2^), 1,068 (65.8%) (95% CI 63.5–68.1) were normal weight (BMI 18.5–24.9 kg/m^2^), 412 (25.4%) (95% confidence interval [CI] 23.3–27.5) were overweight (BMI 25–29.9 kg/m^2^) and 99 (6.1%) (95% CI 5.9–6.3) were obese (BMI ≥ 30 kg/m^2^). Among 1,622 mothers, 95.9% of women had secondary education or higher. About 4 out of 10 mothers were employed (37.5%). As for nationality, there was no difference in pre-pregnancy BMI between Arab mothers, including Emarati, and other non-Arab mothers. About one-third of the mothers gave birth via CS (30.5%). Of the 1,622 children, 6.7% and 9.6% were preterm births and LBW, respectively (Table 1).

**Table 1:** Sociodemographic characteristics of the studied participants in the different pre-pregnancy BMI subgroups in Abu Dhabi, the United Arab Emirates.

**Variables**		**Total**	**Underweight**	**Normal weight**	**Overweight**	**Obese**	
		**(*N* = 1,622)**	**(*N* = 43)**	**(*N* = 1,068)**	**(*N* = 412)**	**(*N* = 99)**	
		**Mean (SD)**	**Mean (SD)**	**Mean (SD)**	**Mean (SD)**	**Mean (SD)**	***p-*value**
Maternal age, years		30.1 (5.2)	28.7 (6.0)	29.3 (4.9)	31.7 (5.0)	32.3 (5.8)	<0.001
Parity		2.2 (1.2)	1.7 (1.1)	2.1 (1.1)	2.7 (1.3)	2.6 (1.4)	<0.001
Gestational age, weeks		39.1 (1.9)	39.5 (1.2)	39.1 (1.9)	39.0 (1.8)	38.7 (2.5)	0.100
Birth weight, grams		3,078 (527)	2,978 (520)	3,064 (514)	3,113 (537)	3,125 (617)	0.180
		***N*** **(%)**	***N*** **(%)**	***N*** **(%)**	***N*** **(%)**	***N*** **(%)**	***p-*** **value**
Maternal age	<30 years	803 (49.5)	27 (62.8)	597 (55.9)	147 (35.7)	32 (32.3)	* <0.001*
	≥30 years	819 (50.5)	16 (37.2)	471 (44.1)	265 (64.3)	67 (67.7)	
Maternal education	< Secondary level	67 (4.1)	5 (11.6)	47 (4.4)	14 (3.4)	1 (1.0)	0.026
	≥Secondary level	1,555 (95.9)	38 (88.4)	1,021 (95.6)	398 (96.6)	98 (99.0)	
Paternal education	< Secondary level	32 (2.0)	0 (0)	22 (2.1)	5 (1.2)	5 (5.1)	0.072
	≥Secondary level	1,590 (98.0)	43 (100)	1,046 (97.9)	407 (98.8)	94 (94.9)	
Nationality	Arab	1,049 (64.7)	23 (53.5)	677 (63.4)	278 (67.5)	71 (71.7)	0.082
	Non-Arab	573 (35.3)	20 (46.5)	391 (36.6)	134 (32.5)	28 (28.3)	
Marital status	Married	1,599 (98.6)	42 (97.7)	1,055 (98.8)	407 (98.8)	95 (96.0)	0.135
	Unmarried	23 (1.4)	1 (2.3)	13 (1.2)	5 (1.2)	4 (4.0)	
Mode of delivery	Vaginal delivery	1,127 (69.5)	31 (72.1)	764 (71.5)	278 (67.5)	54 (54.5)	0.004
	Cesarean delivery	495 (30.5)	12 (27.9)	304 (28.5)	134 (32.5)	45 (45.5)	
Child gender	Male	793 (48.9)	27 (62.8)	509 (47.7)	203 (49.3)	54 (54.5)	0.153
	Female	829 (51.1)	16 (37.2)	559 (52.3)	209 (50.7)	45 (45.5)	
Parity	1st	575 (35.5)	25 (58.1)	432 (40.4)	94 (22.8)	24 (24.2)	<0.001
	≥2	1,047 (64.5)	18 (41.9)	636 (59.6)	318 (77.2)	75 (075.8)	
Maternal employment	Housewife	1,013 (62.5)	28 (65.1)	677 (63.4)	254 (61.7)	54 (54.5)	0.350
	Employed	609 (37.5)	15 (34.9)	391 (36.6)	158 (38.3)	45 (45.5)	
Family income rating	< good	102 (6.3)	3 (7.0)	58 (5.4)	28 (6.8)	13 (13.1)	0.024
	≥good	1,520 (93.7)	40 (93.0)	1,010 (94.6)	384 (93.2)	86 (86.9)	
Initiation of breastfeeding	Early	1,015 (62.6)	25 (581)	668 (62.5)	262 (63.6)	60 (60.6)	0.871
	Delayed	607 (37.4)	18 (41.9)	400 (37.5)	150 (36.4)	39 (39.4)	
Gestational age at delivery	Term (≥37 weeks)	1,514 (93.3)	41 (95.3)	997 (93.4)	384 (93.2)	92 (92.9)	0.956
	Preterm (<37 weeks)	108 (6.7)	2 (4.7)	71 (6.6)	28 (6.8)	7 (7.1)	
Child birth weight at delivery	Normal and large birth weight (≥2,500 grams)	1,467 (90.4)	37 (86.0)	962 (90.1)	380 (92.2)	88 (88.9)	0.402
	Low birth weight (<2,500 grams)	155 (9.6)	6 (14.0)	106 (9.9)	32 (7.8)	11 (11.1)	

In multi-nominal logistic regression analysis, mothers with advanced maternal age (AMA) (≥35 years) were more likely to be overweight odds ratio [OR] = 1.93 (95% CI 1.52–2.3) and obese OR = 2.15 (95% CI 1.32–3.39). Mothers with lower family income were more likely to be obese OR = 2.72 (95% CI 1.44–5.93). Mothers with high parity (≥2) were more likely to be overweight OR = 1.91 (95% CI 1.49–2.50) and obese OR = 1.76 (95% CI 1.06–2.92) and less likely to be underweight OR = 0.52 (95% CI 0.27–0.94). Obese mothers were more likely to deliver via CS OR = 1.95 (95% CI 1.27–2.96). Other adverse pregnancy outcomes such as preterm birth and LBW were not associated with abnormal BMI (i.e., underweight, overweight, and obesity).

Maternal education among the studied participants was not associated with BMI abnormality (Table 2).

**Table 2:** Factors associated with underweight, overweight and obesity among studied participants in Abu Dhabi, the United Arab Emirates using multinomial logistic analysis.

**Variables**	**Underweight**		**Overweight**		**Obese**	
	**OR (95% CI)**	***p-*value**	**OR (95% CI)**	***p-*value**	**OR (95% CI)**	***p-*value**
Maternal age ≥35 vs. <35 years	0.99 (0.53–1.98)	0.992	1.93 (1.52–2.3)	<0.001	2.15 (1.32–3.39)	0.001
Maternal education ≥ secondary vs. < secondary	0.44 (0.14–1.02)	0.058	0.99 (0.55–1.87)	0.970	3.09 (0.45–22.97)	0.271
Family income rating < good vs. ≥ good	1.32 (0.45–4.37)	0.675	1.34 (0.86–2.12)	0.255	2.72 (1.44–5.93)	0.002
Parity≥ 2 order vs. parity 1	0.52 (0.27–0.94)	0.037	1.91 (1.49–2.50)	<0.001	1.76 (1.06–2.92)	0.024
Mode of delivery cesarean delivery vs. vaginal delivery	0.92 (0.48–1.95)	0.956	1.15 (0.92–1.48)	0.263	1.95 (1.27–2.96)	0.002

OR, Odd Ratio; CI, Confidence Interval. The reference category is the normal weight.

## Discussion

To the best of the authors' knowledge, this is the first comprehensive study on the epidemiology of pre-pregnancy BMI in Abu Dhabi, the UAE. Therefore, this work will be considered as an additional value to the few existing data published regarding pre-pregnancy BMI in the UAE (30). The main findings of current works were estimating the prevalence of pre-pregnancy BMI subgroups and exploring the possible associated factors (e.g., age, family income, parity, and CS) with abnormal pre-pregnancy BMI subgroups. More than one-third (34.2%) of the total participants (1,622) had abnormal pre-pregnancy BMI, i.e., underweight (2.7%), overweight (25.7%), and obesity (6.1%).

Compared to the recently published work in the UAE reporting a 59.4% pre-pregnancy BMI ≥25 kg/m^2^ (30), this study revealed less prevalence of overweight/obesity (31.8%). The discrepancies in the prevalence could be attributed to many reasons, such as the small sample size of the former study, which included only Emirates and Arab mothers' nationalities (30), unlike this study. A similar prevalence of normal pre-pregnancy BMI (63.5%) was reported by several studies, e.g., in Canada (65.8%) (15) and in France (64.2%) (23); however, the difference is within the abnormal pre-pregnancy BMI subcategories. A study in Saudi Arabia reported 29.7% normal BMI and a very high prevalence of pre-pregnancy overweight and obesity (31). Similar results of a high prevalence of pre-pregnancy overweight were observed in Qatar and Lebanon (32).

In this work, maternal education was positively associated with pre-pregnancy BMI in the univariate analysis. However, this association was not identified in the logistic regression analysis. The literature is contradictory regarding the association between maternal education and abnormal pre-pregnancy BMI (33, 34). For example, high maternal education was associated with overweight/obesity in Bangladesh (33). Whereas in Taiwan, it was the opposite (34). The influence of education on pre-pregnancy BMI could be attributed to the quality of education more than the class attended (< secondary or ≥secondary level).

In addition, mothers with lower family income were more likely to be obese OR = 2.72 (95% 1.44–5.93).

The literature results vary regarding the association between family income and obesity, some results go with high family income associated with obesity (32), and others (34) oppose this results. In support of this results, the strong correlation between lower-income and the risk of obesity was revealed in the literature even by meta-analysis (35). Besides, the coexistence of malnutrition, undernutrition, and over-nutrition, and its huge negative impact on the health and economy is common in low- and middle-income countries (36, 37).

Mothers with high parity (≥2) were also more likely to be overweight OR = 1.91 (95% 1.49–2.50), obese OR = 1.76 (95% 1.06–2.92), and less likely to be underweight OR = 0.52 (95% 0.27–0.94). Many studies, including the studied country, documented the strong correlation between higher parity and obesity (30, 34, 38, 39). A study in Denmark estimated that the average gained 0.62 BMI units after every additional birth (38). Therefore, increasing parity will be associated with increased overweight/obesity. Even the factors related to abnormal pre-pregnancy BMI are correlated, i.e., AMA with high parity (40) and AMA with CS (18). This results showed among mothers with high birth order, 1,047, 640 (61.1%) were older and high parity. Interestingly, high parity influences the mode of delivery in many ways. For example, high parity was reported to be associated with anemia (41) and CS (42).

The study revealed that obese mothers were almost twice as likely to deliver via cesarean section OR = 1.95 (95% 1.27–2.96). Furthermore, obesity decreased the chance of successful vaginal birth after cesarean section (VBAC) (43, 44). Many authors have documented the negative impact of obesity on pregnancy outcomes, including CS (34, 45–47). Besides, CS is correlated with adverse effects such as maternal mortality, perinatal morbidity, and mortality (48). In many settings, including the UAE, pre-pregnancy BMI was a common denominator in the etiology of excessive gestational weight gain (30, 34). In addition, pre-pregnancy obesity and greater weight gain independently increase the risk of CS (34, 49). Therefore, programs to reduce pre-pregnancy obesity might reduce the high rates of CS and good pregnancy outcomes. For instance, a study in Italy documented reducing the most adverse pregnancy outcomes when decreasing pre-pregnancy BMI (50).

Unlike this results, abnormal pre-pregnancy BMI, such as obesity and underweight, was found to be associated with some poor perinatal outcomes, e.g., LBW (13, 46), preterm birth (34, 46, 51), and poor breastfeeding practices, e.g., breastfeeding initiation and duration (52, 53), and gestational diabetes mellitus (GDM) (54). The variations between the current and previous results indicate the complexity of influencing abnormal pre-pregnancy BMI on maternal and perinatal and early child health. Such variations should encourage researchers to conduct more research to explore this complexity by estimating the magnitude of these abnormal BMI groups, finding the associated impacts, and motivating the involved parties to take appropriate actions to improve maternal and child health.

To our knowledge, this is the first comprehensive study tackling pre-pregnancy BMI epidemiology in Abu Dhabi, the UAE. This study revealed valuable information from a large sample of the community, which healthcare planners can use to improve maternal and child health by addressing the maintenance of a healthy BMI before, during, and after delivery to achieve good pregnancy outcomes. Hence, the results will help the health authorities in the UAE to ensure that they are providing the best guidance and care for pregnant women. In addition, the results underscore the need for culture-specific intervention programs to promote healthy body weight in women of childbearing age. More accurate and precise policies would be formulated on weight gain recommendations and weight monitoring during pregnancy.

However, our study has some limitations which are (a) the recall bias as the data collection included children up to 2 years old, (b) possible bias on these results due to missing data, and (c) not including the possible impact of BMI on maternal and child morbidity and mortality.

## Conclusions

This study provides valuable information that can be used by policymakers as a tool to improve maternal and child health in order to maintain a normal BMI for a healthy pregnancy outcome. Additionally, there is a need to strengthen the available healthcare system for the overall prevention of obesity through primary care as a leading system and establish a structured network of specialized obesity centers. Moreover, developing guidelines on weight gain for optimizing pregnancy and neonatal outcomes will provide an excellent communication tool for healthcare experts, mothers, and children. Finally, additional intervention research is needed, including designed lifestyle programs to improve pregnancy outcomes.

## Data availability statement

The raw data supporting the conclusions of this article will be made available by the authors, without undue reservation.

## Ethics statement

The studies involving human participants were reviewed and approved by (ZU17_006_F) the Research Ethics Committee at Zayed University, the UAE. The patients/participants provided their written informed consent to participate in this study.

## Author contributions

ZT designed the study and recruited the participants. ZT and AH analyzed the data and wrote the manuscript. DP contributed to the design of the study, data collection, and manuscript writing. All authors read, critically revised, and approved the final manuscript.

## Funding

This research was funded by the research incentive fund (R17042) from the Research Office at Zayed University.

## Conflict of interest

The authors declare that the research was conducted in the absence of any commercial or financial relationships that could be construed as a potential conflict of interest.

## Publisher's note

All claims expressed in this article are solely those of the authors and do not necessarily represent those of their affiliated organizations, or those of the publisher, the editors and the reviewers. Any product that may be evaluated in this article, or claim that may be made by its manufacturer, is not guaranteed or endorsed by the publisher.

## Abbreviations

OR: Odds Ratios
BMI: Body Mass Index
CI: Confidence Interval
LBW: Low Birth Weight
CS: Cesarean Section
SD: Standard Deviation
SPSS: Statistical Package for Social Science
UAE: United Arab Emirates
WHO: World Health Organization.
